# Pulmonary *Mycobacterium shigaense* Infection with Hemoptysis: Two Surgically Resected Case Reports

**DOI:** 10.70352/scrj.cr.26-0092

**Published:** 2026-06-02

**Authors:** Naoko Ose, Kaoru Fukuyama, Yusuke Sugiura, Eiichi Morii, Yasushi Shintani

**Affiliations:** 1 Department of General Thoracic Surgery, The University of Osaka Graduate School of Medicine, Suita, Osaka, Japan; 2 Department of Pathology, The University of Osaka Graduate School of Medicine, Suita, Osaka, Japan

**Keywords:** *Mycobacterium shigaense*, nontuberculous mycobacterium, hemoptysis, pulmonary infection, surgical resection

## Abstract

**INTRODUCTION:**

*Mycobacterium shigaense (M. shigaense)* is a rare nontuberculous mycobacterium (NTM) first reported in Japan in 2012. Only a few cases of pulmonary infection have been described, all treated medically. No surgically resected cases or cases presenting with hemoptysis have been reported to date.

**CASE PRESENTATION:**

We report 2 immunocompetent patients with pulmonary *M. shigaense* infection presenting with persistent hemoptysis who were successfully treated surgically. Case 1 was a 67-year-old woman with daily hemoptysis caused by a nodular lesion in the right middle lobe. Despite long-term antimicrobial therapy, hemoptysis worsened, and a robot-assisted right middle lobectomy was performed. Hemoptysis resolved immediately after surgery, and no recurrence was observed during 1 year of follow-up. Case 2 was a 68-year-old man with repeated moderate hemoptysis caused by a cavitary lesion in the left upper lobe. Robot-assisted surgery was attempted but converted to thoracotomy due to severe adhesions, and left upper division segmentectomy was performed. Hemoptysis was resolved completely after surgery. In both cases, *M. shigaense* was identified by culture and genomic analysis, and histopathology showed epithelioid granulomas with caseous necrosis.

**CONCLUSIONS:**

Pulmonary *M. shigaense* infection can cause clinically significant hemoptysis. Surgical resection combined with antimicrobial therapy was effective for symptom control in both cases. Surgery should be considered for localized disease refractory to medical treatment.

## Abbreviations

HE
: hematoxylin and eosin
MAC
: *Mycobacterium avium* complex
*M. shigaense*
: *Mycobacterium shigaense*
NTM
: nontuberculous mycobacteria

## INTRODUCTION

NTM are environmental organisms found in water and soil, and approximately 250 species have been reported to date.^1)^ The prevalence of NTM species varies by region according to environmental background and climate; however, the global incidence of NTM infections is increasing worldwide.^2)^ MAC is the most common causative organism globally, although the predominant species differ by region. Because antimicrobial susceptibility and prognosis vary among NTM species, precise species identification is essential. In addition, rare region-specific species exist, and genomic analysis may be required for accurate identification in some cases.

*M. shigaense* was first identified in Japan in 2009 and formally reported in 2012 as a novel NTM species.^3)^ Reported cases remain extremely rare and are limited to East Asia. To date, cutaneous infections,^3–5)^ disseminated infections,^6,7)^ and pulmonary infections^8,9)^ have been described. Only 3 cases of pulmonary infection have been reported, all of which were treated with medical therapy alone. No surgically resected cases have previously been reported, and no cases presenting with hemoptysis have been described.

Here, we report the first 2 surgically treated cases of pulmonary *M. shigaense* infection in immunocompetent patients presenting with hemoptysis and discuss the clinical features in comparison with previously reported cutaneous and disseminated forms.

## CASE PRESENTATION

### Case 1

**Fig. 1:**
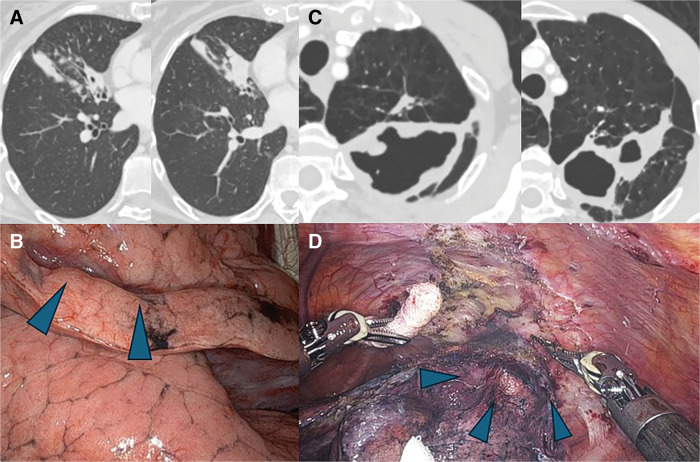
Findings of chest CT and intraoperative findings. (**A**) Chest CT revealed several nodules and bronchiectasis in the right middle lobe in Case1. (**B**) There were no adhesions, and the lesion corresponding to the suspected bleeding source was revealed with pleural indentation and neovascularization on the pleural surface overlying the lesion in Case 1 (arrowheads). (**C**) Chest CT revealed a cavity in the left upper lobe in Case 2. (**D**) There were ridge adhesions between the parietal pleura and aorta. The blue arrowheads indicate the cavity lesion in Case 2.

A 67-year-old woman presented with hemoptysis. Chest CT revealed a small nodule with bronchiectasis in segment 5 of the right middle lobe (**Fig. 1A**). Bronchoscopic culture identified *M. shigaense*. Antimicrobial therapy based on drug susceptibility testing was initiated; however, over a 2-year follow-up period, the frequency of hemoptysis gradually increased, eventually occurring daily.

The lesion in the right middle lobe was considered the source of bleeding, and robot-assisted right middle lobectomy was performed. Intraoperatively, mild pleural adhesions were noted, and a nodule was identified in segment 5 (**Fig. 1B**). Although development of the bronchial artery was observed, there were no significant hilar adhesions, and the lesion was resected using a minimally invasive approach. Hemoptysis ceased immediately after surgery, and the postoperative course was uneventful.

**Fig. 2:**
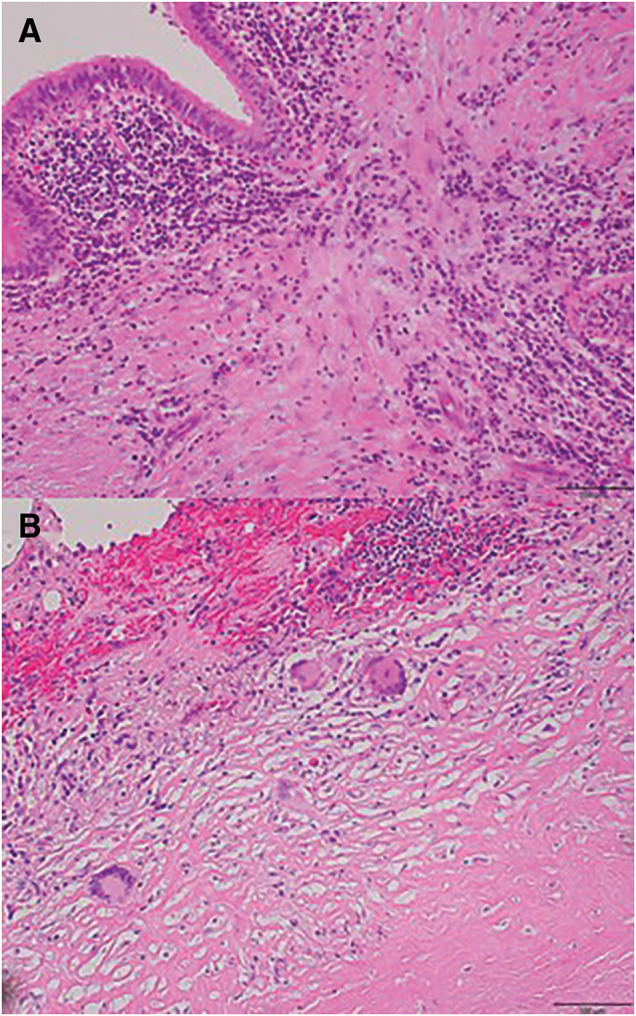
Histological findings of the resected specimen. (**A**) HE staining revealed that inflammatory cell infiltration was present around the bronchi in Case 1. (**B**) HE staining revealed an accumulation of Langerhans giant cells and the presence of epithelioid granulomas in Case 2. HE, hematoxylin and eosin

Macroscopically, the resected specimen showed a white nodule with caseous necrosis. Histopathological examination demonstrated inflammatory cell infiltration around the bronchi (**Fig. 2A**). Postoperatively, combination chemotherapy with rifampicin, ethambutol, and clarithromycin was continued; however, treatment was discontinued after 9 months because of adverse effects. More than 1 year after surgery, no recurrence of hemoptysis or radiological deterioration has been observed.

### Case 2

A 68-year-old man presented with hemoptysis. Chest CT showed a cavitary lesion in segment 1+2c of the left upper lobe (**Fig. 1C**). He experienced repeated episodes of moderate hemoptysis requiring hospitalization. The cavity in the left upper lobe was identified as the source of bleeding, and surgical treatment was indicated.

Robot-assisted left upper division segmentectomy was initially attempted; however, dense adhesions were encountered (**Fig. 1D**), particularly between the cavity wall and the aortic arch. During the operation, dense inflammatory adhesions, particularly along the mediastinal side of the aorta, made safe dissection difficult, and adequate visualization could not be obtained with the robotic approach. Consequently, conversion to thoracotomy was required, and left upper division segmentectomy was completed. Hemoptysis disappeared immediately after surgery, and the postoperative course was uneventful.

Sputum submitted for genomic analysis and culture from the resected specimen both identified *M. shigaense*. Histopathological findings demonstrated white nodules with epithelioid granulomas and Langerhans giant cells (**Fig. 2B**). Based on drug susceptibility testing, antimicrobial therapy with rifampicin, ethambutol, and clarithromycin was initiated and is ongoing. No recurrence of hemoptysis or radiological progression has been observed.

## DISCUSSION

*M. shigaense* is an emerging NTM species first reported in Japan in 2012.^3)^ It belongs to Runyon group II, which includes slow-growing, scotochromogenic mycobacteria, and is closely related to *Mycobacterium simiae*.^10)^ To date, only 10 cases have been reported worldwide (**Table 1**), with most cases identified in the area surrounding Shiga Prefecture, Japan. The 2 patients in the present report lived in Osaka Prefecture, which is geographically distant from Shiga; however, both regions are part of the Lake Biwa watershed, suggesting a possible geographic association.

**Table 1:** Reported cases of *Mycobacterium shigaense*

No.	Year	Author	Age	Sex	Clinical type	Immunological background	Lesion organ	Symptoms	Treatment	NTM course	Prognosis
1	2012	Nakanaga et al.^3)^	55	M	Cutaneous	Hodgkin’s disease	Skin	Skin lesion	C, I	Improved	Died of Hodgkin’s disease
2	2013	Cui et al.^4)^	56	F	Cutaneous	Immunocompetent	Skin	Skin lesion	R, C moxifloxacin	Improved	Alive
3	2016	Koizumi et al.^5)^	40	M	Cutaneous	AIDS	Subcutaneous lymph node	Low-grade fever, lymphadenopathy	RBT, E, I	Improved	Alive
4	2016	Naito et al.^6)^	71	M	Disseminated	Castleman’s disease with immunosuppressants	Skin, bone marrow, lung, liver, spleen	Fever, skin lesions	RC, LVFX	Worsen	Died of pneumonia
5	2018	Hatsuse et al.^7)^	73	M	Disseminated	Lymphoma	Skin, spleen	Fever, skin lesions	C, LVFX	Improved	Alive
6	2019	Fukano et al.^8)^	58	M	Pulmonary	Immunocompetent	Lung with NB type	None	R, E, C	Improved	Alive
7	2020	Yoshida et al.^9)^	88	M	Pulmonary	Immunocompetent	Lung with FC type	Cough, fever	R, E, C	Improved	Alive
8			78	F	Pulmonary	Immunocompetent	Lung with NB type	None	None	Improved	Alive
9	2025	Our case	72	F	Pulmonary	Immunocompetent	Lung with NB type	Hemoptysis	Surgery + R, E, C	Improved	Alive
10			74	M	Pulmonary	Immunocompetent	Lung with FC type	Hemoptysis	Surgery + R, E, C	Improved	Alive

C, clarithromycin; E, ethambutol; F, female; FC type, fibrocavitary type; I, isoniazid; LVFX, levofloxacin; M, male; NB type, nodular bronchiectatic type; NTM, nontuberculous mycobacteria; R, rifampicin; RBT, rifabutin

Reported infections can be classified into 3 clinical types: cutaneous, disseminated, and pulmonary. Cutaneous infection cases were successfully treated with antimicrobial therapy.^3–5)^ Disseminated infection has been reported in 2 immunocompromised patients, both with widespread organ involvement.^6,7)^ Pulmonary infection was first reported in 2020 by Fukano et al.,^8)^ followed by 2 additional cases reported by Yoshida et al.^9)^ Including the present cases, only 5 cases of pulmonary *M. shigaense* infection have been reported. All patients were immunocompetent and exhibited either fibrocavitary or nodular–bronchiectatic patterns.

Previously reported pulmonary cases were asymptomatic and presented only with abnormal chest imaging findings; all were treated successfully with medical therapy alone. In contrast, both of our patients presented with moderate hemoptysis that was refractory to medical treatment. Hemoptysis significantly impairs QOL and may be life-threatening when massive. Therefore, hemoptysis is considered an indication for surgical treatment even in NTM infections, according to the ATS/ERS/ESCMID/IDSA guideline.^11)^ In the present cases, hemoptysis persisted despite appropriate antimicrobial therapy, and the responsible lesions were localized, supporting the decision to perform surgical resection. Although nonsurgical interventional treatment such as bronchial artery embolization may be considered for hemoptysis, especially for temporary hemostasis, its role in achieving definitive control of localized infectious lesions remains limited. Therefore, surgical resection was considered an appropriate treatment option in our cases.

Macroscopically, there was no evidence of chest wall neovascularization through adhesions, as typically seen in pulmonary aspergillosis. Instead, marked development of bronchial arteries was observed. Histopathological findings varied slightly between the 2 cases; however, granulomatous inflammation consistent with NTM infection was observed in both, supporting the diagnosis. The mechanism of hemoptysis was presumed to be associated with chronic inflammation causing dilation, tortuosity, and rupture of the bronchial arteries, or possibly destruction of pulmonary arteries like a Rasmussen aneurysm in tuberculosis.

These findings suggest that *M. shigaense* is a species capable of causing hemoptysis in pulmonary infection. Given the rarity of this organism, some cases may have remained unidentified or misidentified to date. Therefore, recognition of this clinical presentation is important for appropriate diagnosis and management.

As with other NTM infections, antimicrobial chemotherapy is the mainstay of treatment for pulmonary *M. shigaense* infection. However, in both of our cases, resection of the main lesion resulted in complete resolution of symptoms. Therefore, the surgical indications for pulmonary *M. shigaense* infection may be considered similar to those for MAC, as recommended in the ATS/ERS/ESCMID/IDSA guideline.^11)^ In carefully selected cases, minimally invasive surgery, including robot-assisted approaches, may be a feasible treatment option. At the same time, surgery for infectious pulmonary lesions requires careful patient selection because inflammatory adhesions, tissue fragility, intraoperative bleeding, prolonged air leak, postoperative infectious complications, and possible conversion to thoracotomy should be taken into consideration.

## CONCLUSIONS

*M. shigaense* is a rare NTM species that can present with diverse clinical manifestations, including cutaneous, pulmonary, and disseminated forms. Pulmonary infection may cause hemoptysis, as observed in our cases. Combined medical therapy and surgical resection of the main lesion were effective for controlling hemoptysis. Surgical intervention should be considered in cases with localized disease and persistent hemoptysis.

## DECLARATIONS

### Availability of data and materials

The dataset supporting the conclusions of this article is included within the article

## References

[1] LPSN. List of prokaryotic names with standing in nomen clature. http://www.bacterio.net/index.html. 10.1093/nar/gkt1111 PMC3965054 24243842

[2] Kendall BA, Winthrop KL. Update on the epidemiology of pulmonary nontuberculous mycobacterial infections. Semin Respir Crit Care Med 2013; 34: 87–94. 23460008 10.1055/s-0033-1333567

[3] Nakanaga K, Hoshino Y, Wakabayashi M, et al. Mycobacterium shigaense sp. nov., a novel slowly growing scotochromogenic mycobacterium that produced nodules in an erythroderma patient with severe cellular immunodeficiency and a history of Hodgkin’s disease. J Dermatol 2012; 39: 389–96. 21955184 10.1111/j.1346-8138.2011.01355.x

[4] Cui P, Vissa V, Li W, et al. Cutaneous Mycobacterium shigaense infection in immunocompetent Woman, China. Emerg Infect Dis 2013; 19: 819–20. 23697461 10.3201/eid1905.121022 PMC3647498

[5] Koizumi Y, Shimizu K, Shigeta M, et al. Mycobacterium shigaense causes lymph node and cutaneous lesions as immune reconstitution syndrome in an AIDS patient: the third case report of a novel strain non-tuberculous mycobacterium. Intern Med 2016; 55: 3375–81. 27853087 10.2169/internalmedicine.55.6996 PMC5173512

[6] Naito D, Mizumoto C, Takeoka T, et al. A case of disseminated Mycobacterium shigaense infection. Nihon Naika Gakkai Zasshi 2016; 105: 717–22. 27491265 10.2169/naika.105.717

[7] Hatsuse M, Daikoku Y, Taminishi Y, et al. Possible contribution of disseminated Mycobacterium shigaense infection to development of splenic marginal zone lymphoma. Rinsho Ketsueki 2018; 59: 878–83. (in Japanese) 30078797 10.11406/rinketsu.59.878

[8] Fukano H, Hiranuma O, Matsui Y, et al. The first case of chronic pulmonary *Mycobacterium shigaense* infection in an immunocompetent patient. New Microbes New Infect 2019; 33: 100630. 31908785 10.1016/j.nmni.2019.100630 PMC6940610

[9] Yoshida S, Iwamoto T, Kobayashi T, et al. Two new cases of pulmonary infection by Mycobacterium shigaense, Japan. Emerg Infect Dis 2020; 26: 2728–32. 33079053 10.3201/eid2611.200315 PMC7588508

[10] Fukano H, Yoshida M, Kazumi Y, et al. Mycobacterium shigaense sp. nov., a slow-growing, scotochromogenic species, is a member of the Mycobacterium simiae complex. Int J Syst Evol Microbiol 2018; 68: 2437–42. 29939124 10.1099/ijsem.0.002845

[11] Daley CL, Iaccarino JM, Lange C, et al. Treatment of nontuberculous mycobacterial pulmonary disease: an official ATS/ERS/ESCMID/IDSA clinical practice guideline. Eur Respir J 2020; 56: 2000535. 32636299 10.1183/13993003.00535-2020 PMC8375621

